# Testing Practices, Interpretation, and Diagnostic Evaluation of Iron Deficiency Anemia by US Primary Care Physicians

**DOI:** 10.1001/jamanetworkopen.2021.27827

**Published:** 2021-10-01

**Authors:** Andrew J. Read, Akbar K. Waljee, Jeremy B. Sussman, Hardeep Singh, Grace Y. Chen, Sandeep Vijan, Sameer D. Saini

**Affiliations:** 1 Department of Internal Medicine, University of Michigan, Ann Arbor; 2 Institute for Healthcare Policy and Innovation, University of Michigan, Ann Arbor; 3 Veterans Affairs Health Services Research and Development, Center for Clinical Management Research, Ann Arbor, Michigan; 4 Center for Innovations in Quality, Effectiveness and Safety, Michael E. DeBakey Veterans Affairs Medical Center and Baylor College of Medicine, Houston, Texas

## Abstract

**Importance:**

Recognition of iron deficiency anemia (IDA) is important to initiate timely evaluation for gastrointestinal tract cancer. Retrospective studies have reported delays in diagnostic evaluation of IDA as a common factor associated with delayed diagnosis of colorectal cancer.

**Objective:**

To assess how US primary care physicians (PCPs) approach testing for anemia, interpret iron laboratory studies, and refer patients with IDA for gastrointestinal endoscopy.

**Design, Setting, and Participants:**

This survey study, conducted in August 2019, included members of the American College of Physicians Internal Medicine Insiders Panel, a nationally representative group of American College of Physicians membership, who self-identified as PCPs. Participants completed a vignette-based survey to assess practices related to screening for anemia, interpretation of laboratory-based iron studies, and appropriate diagnostic evaluation of IDA.

**Main Outcomes and Measures:**

Descriptive statistics based on survey responses were evaluated for frequency of anemia screening, correct interpretation of iron laboratory studies, and proportion of patients with new-onset IDA referred for gastrointestinal tract evaluation.

**Results:**

Of 631 PCPs who received an invitation to participate in the survey, 356 (56.4%) responded and 31 (4.9%) were excluded, for an adjusted eligible sample size of 600, yielding 325 completed surveys (response rate, 54.2%). Of the 325 participants who completed surveys, 180 (55.4%) were men; age of participants was not assessed. The mean (SD) duration of clinical experience was 19.8 (11.2) years (range, 1.0-45.0 years). A total of 250 participants (76.9%) screened at least some patients for anemia. Interpretation of iron studies was least accurate in a scenario of a borderline low ferritin level (40 ng/mL) with low transferrin saturation (2%); 86 participants (26.5%) incorrectly responded that this scenario did not indicate IDA, and 239 (73.5%) correctly identified this scenario as IDA. Of 312 participants, 170 (54.5%) recommended bidirectional endoscopy (upper endoscopy and colonoscopy) for new IDA for women aged 65 years; of 305 respondents, 168 (55.1%) recommended bidirectional endoscopy for men aged 65 years.

**Conclusions and Relevance:**

In this survey study, US PCPs’ self-reported testing practices for anemia suggest overuse of screening laboratory tests, misinterpretation of iron studies, and underuse of bidirectional endoscopy for evaluation of new-onset IDA. Both misinterpretation of iron studies and underuse of bidirectional endoscopy can lead to delayed diagnosis of gastrointestinal tract cancers and warrant additional interventions.

## Key Points

**Question:**

How do US primary care physicians approach testing for anemia, interpretation of iron studies, and diagnostic evaluation for iron deficiency anemia?

**Findings:**

In this survey study of 325 US primary care physicians, 76.9% of respondents self-reported screening patients for anemia, 73.5% correctly diagnosed iron deficiency anemia, and 54.5% recommended bidirectional endoscopy for new-onset iron deficiency anemia among women aged 65 years (55.1% for men aged 65 years).

**Meaning:**

Screening for anemia was commonly self-reported by primary care physicians despite the absence of guidelines recommending routine anemia screening, and bidirectional endoscopy was underused to evaluate new-onset iron deficiency anemia.

## Teaser

This survey study uses hypothetical patient and clinical scenarios to assess how US primary care physicians (PCPs) approach screening for iron deficiency anemia, interpret iron laboratory studies, and refer patients with iron deficiency anemia for gastrointestinal endoscopy.

## Introduction

Iron deficiency anemia (IDA) is a classic early diagnostic sign of gastrointestinal (GI) tract malignant neoplasm.^1,2^ Between 1% and 10% of adult patients with IDA may have undiagnosed GI tract cancer.^3,4^ Prompt and thorough evaluation of IDA is essential. However, retrospective studies have reported that delays in diagnostic evaluation of IDA are common^5,6^ and can lead to a delayed diagnosis of colorectal cancer.^7,8^ This problem is of increasing importance given the increasing incidence of colorectal cancer among younger patients outside the age range for which routine colorectal cancer screening is recommended, for whom IDA may be the first diagnostic sign of malignant neoplasm.^9,10^

Clinical practices related to identification and evaluation of IDA likely vary substantially, with at least 30 different guidelines from 10 specialty societies.^11,12,13,14^ However, variation related to testing practices, interpretation of laboratory test results, and diagnostic evaluation of IDA among physicians is not well studied even though these factors may be associated with diagnostic delays, missed cancer diagnoses, and low-value care. For instance, because of a low diagnostic yield, anemia screening is not recommended for adults except during pregnancy, and recommendations about screening during pregnancy are mixed.^15,16,17^ Inappropriate screening may be associated with health care waste and potential unintended harm (eg, additional downstream diagnostic tests). Inaccurate test interpretation of iron studies (eg, serum ferritin level and iron saturation) can also lead to medical error, with misclassification of patients’ iron level. Interpretation can be complicated because the ferritin level may be elevated in the context of an inflammatory process, potentially masking underlying iron deficiency.^2,18^ Guidelines consistently recommend that men with IDA and postmenopausal women, in the absence of obvious causes, should be evaluated for potential occult GI tract blood loss with colonoscopy and esophagogastroduodenoscopy (EGD).^11,12^ However, older guidelines, which predated the increasing incidence of colorectal cancer among younger patients observed over the past decade, recommended against initial endoscopic evaluation for premenopausal women with IDA given the more common cause of iron deficiency secondary to menstrual blood loss.^11^ Together, these issues introduce ambiguity and complexity to the evaluation of IDA.

Our objective was to understand how primary care physicians (PCPs) approach testing and evaluation of patients for IDA. To do this, we conducted a survey study consisting of a vignette-based assessment of PCPs to examine their practices related to IDA testing and evaluation for male and female patients of different ages. A better understanding of current PCP evaluation and referral patterns for GI endoscopy in the context of IDA may reveal potential opportunities to intervene and promote earlier diagnosis of GI tract cancers.

## Methods

### Study Population and Survey Distribution

For this survey study, we conducted a national online survey of internal medicine PCPs in August 2019 using the American College of Physicians (ACP) Internal Medicine Insiders Panel. The ACP Insiders Panel is a nationally representative group of internal medicine physicians selected through stratified random sampling as representative of the ACP’s membership as a whole.^19^ The ACP is the largest internal medicine specialty organization, with 163 000 members. Potentially eligible participants (identified as PCPs who had completed training within the ACP Insiders Panel demographics profile) received an email invitation to complete the electronic survey, and those who completed the survey received points that could be redeemed for Amazon.com gift cards. Two email reminders were sent during a 2-week eligibility period. All respondents had to meet the following inclusion criteria: (1) practice outpatient primary care medicine, (2) practice primarily general internal medicine and not a subspecialty, and (3) have completed residency training. The study was deemed exempt from the requirement for informed consent by the University of Michigan institutional review board owing to minimal potential harm related to completion of this survey study because no patient- or physician-identifiable information was collected. The study followed the American Association for Public Opinion Research (AAPOR) reporting guideline.

### Questionnaire Design

We developed a survey to assess current PCP practices for the testing and evaluation of IDA. Clinical vignettes provided with the survey questions were used to address 3 sequential domains to elicit physicians’ clinical practices and decision-making processes^20^: (1) the practice of obtaining a complete blood count (CBC) for asymptomatic healthy individuals (ie, screening for anemia), (2) PCP ordering and interpretation of iron laboratory studies, and (3) evaluation and management of IDA and referral for GI endoscopy (colonoscopy and/or EGD). For screening, 5 scenarios were used to understand testing practices for 5 hypothetical asymptomatic individuals presenting as new patients: a 35-year-old man, a 35-year-old woman (not pregnant), a 35-year-old pregnant woman, a 65-year-old man, and a 65-year-old woman. For interpretation of iron laboratory studies, a series of 4 combinations of ferritin level and transferrin saturation were presented: low transferrin saturation (6%) and a low ferritin level (11 ng/mL) (to convert to μg/L, multiply by 1.0), normal transferrin saturation (30%) and normal ferritin level (150 ng/mL), normal transferrin saturation (25%) and low ferritin level (6 ng/mL), and low transferrin saturation (2%) and normal to low ferritin level (40 ng/mL). Respondents selected whether the scenario represented anemia due to iron deficiency or another cause.

Four hypothetical patient scenarios were presented for evaluation of management of newly diagnosed IDA to assess use of EGD and colonoscopy: a 35-year-old man, a 35-year-old woman (not pregnant), a 65-year-old man, and a 65-year-old woman. We also developed and included 2 questions regarding general attitudes about diagnostic testing: 1 focused on watching and waiting and 1 focused on laboratory tests and diagnostic procedures.^21^ The questionnaire included a subset of questions related to knowledge and application of the 2018 American Cancer Society Colorectal Cancer Screening Guideline,^22^ and these questions were analyzed separately.^23^ The questionnaire was developed in consultation with a survey development team and iteratively modified based on feedback from 10 PCPs at the University of Michigan, Ann Arbor. The final version of the questionnaire is available in the eAppendix in the Supplement.

### Statistical Analysis

Survey responses are summarized using proportions, and summary statistics are reported for continuous variables. A 2-sided *P* < .05 was considered statistically significant. We used a χ^2^ test for analysis of the categorical variables. Statistical analysis was performed using SAS, version 9.4 (SAS Institute). Graphs were produced using GraphPad Prism, version 8.0 (GraphPad Software).

## Results

### Respondent Characteristics

The survey was distributed electronically via email invitation to 633 individuals on the ACP Insiders Panel in August 2019 who met prescreening criteria as PCPs (according to the ACP Insiders Panel demographics profile); 2 could not be reached. Of 631 PCPs who received an invitation, 356 (56.4%) responded and 31 (4.9%) were excluded, for an adjusted eligible sample size of 600, yielding 325 completed surveys (response rate, 54.2%). Of the 325 respondents who completed the survey, 180 (55.4%) listed their gender as male and 315 (96.9%) were board certified in internal medicine; age of respondents was not assessed (Table). Respondents represented a wide variation of clinical practice experience. Clinical practice duration ranged from 1.0 to 45.0 years (mean [SD], 19.8 [11.2] years); 231 respondents (71.1%) practiced exclusively outpatient primary care, and 120 (36.9%) had an academic affiliation to a medical school. Of 325 physicians, 303 (93.2%) agreed that too many laboratory tests and diagnostic procedures are performed in the US health care system.

**Table.** Characteristics of 325 Respondents to the Survey

Characteristic	Respondents^a^
Time in clinical practice, y	
Mean (range)	19.8 (1.0-45.0)
Median (IQR)	20.0 (10.0-29.0)
Region	
Urban	129 (39.7)
Suburban	167 (51.4)
Rural	29 (8.9)
Gender	
Male	180 (55.4)
Female	131 (40.3)
Prefer not to answer	14 (4.3)
Race and ethnicity	
Asian	80 (24.6)
Black or African American	6 (1.9)
White	193 (59.4)
Other^b^	7 (2.2)
Prefer not to answer	39 (12.0)
Affiliated with a medical school	
Yes	120 (36.9)
No	205 (63.1)
Board-certified in internal medicine	
Yes	315 (96.9)
No	10 (3.1)
Practice setting	
Single-specialty office	125 (38.5)
Multispecialty office	90 (27.7)
Medical school or academic medical center	34 (10.5)
US government^c^	23 (7.1)
Hospital based	21 (6.5)
Free-standing ambulatory care or urgent care center	10 (3.1)
Institution^d^	5 (1.5)
Other	17 (5.2)
Type of practice where most time is spent	
All outpatient	231 (71.1)
Primarily outpatient with some inpatient	75 (23.1)
Primarily inpatient with some outpatient	10 (3.1)
Equal outpatient and inpatient	9 (2.8)
General attitudes toward testing	
Reluctant to watch and wait	221 (68.0)
Overuse of laboratory tests and diagnostic procedures	303 (93.2)

Abbreviation: IQR, interquartile range.

^a^ Data are presented as number (percentage) of respondents unless otherwise indicated.

^b^ Self-identified as other than the listed choices.

^c^ Includes Veterans Affairs and military settings.

^d^ Includes prisons, nursing facilities, and other institutions.

Demographic information from the ACP Insiders Panel was analyzed to compare potential differences between respondents and nonrespondents. Demographic data from August 2019 were available for 454 of the 633 participants (71.7%) who were invited to complete the survey, including 240 who responded to the survey and 214 who did not respond. There were no statistically significant differences between respondents and nonrespondents by geographic region (West, Midwest, Northeast, or South), age group (<40 years, 40-55 years, or >55 years), specialty (general internal medicine, subspecialist, hospitalist, or other), or career stage (graduated medical school >16 years ago or ≤16 years ago). Respondents were more likely than nonrespondents to be White than a member of another racial or ethnic group (race and ethnicity were analyzed as dichotomous variables as collected by the ACP Insiders Panel team based on participant self-report) (146 of 215 White respondents [67.9%] vs 104 of 195 White nonrespondents [53.3%]; *P* = .003) and were more likely than nonrespondents to be male (141 of 236 male respondents with gender data [59.7%] vs 102 of 207 male nonrespondents with gender data [49.3%]; *P* = .03).

### Screening for Anemia

**Figure 1.**
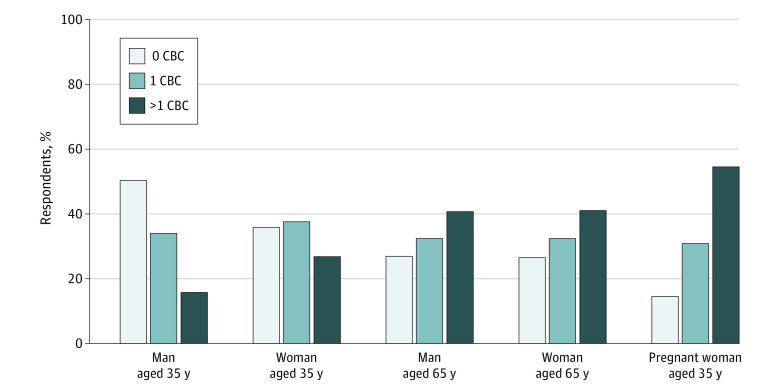
Rates of Obtaining Complete Blood Counts (CBCs) for Detection of Anemia in Asymptomatic Patients by Age, Sex, and Pregnancy Status for Healthy Patients Establishing Care With a Primary Care Physician Data are based on survey responses of 325 primary care physicians reporting recommendations of 1 CBC, repeated CBCs, or no CBC. For pregnant women, there were 220 respondents because primary care physicians who did not provide care to pregnant women were excluded.

Respondents were asked about their practice of testing asymptomatic healthy patients for anemia by obtaining CBCs (screening) in a series of hypothetical scenarios involving 35-year-old or 65-year-old men and women presenting as new patients to their practices. Obtaining screening CBCs was a common practice among respondents; 250 of 325 physicians (76.9%) reported testing at least some asymptomatic patients aged 35 years or 65 years (Figure 1). Of 325 PCPs, 159 (48.9%) reported they would test all these new patients for anemia by obtaining a CBC. Physicians who reported being generally reluctant to recommend watching and waiting were significantly more likely to recommend screening all the groups for anemia (odds ratio [OR], 4.23; 95% CI, 2.53-7.07; *P* < .001). Physicians’ gender, practice region (urban, suburban, or rural), practice type (exclusively outpatient practice vs some inpatient practice), and stage in clinical practice (<5 years or ≥5 years) were not significantly associated with reporting screening. Physicians for whom the primary practice setting was an academic medical center were less likely to recommend obtaining screening CBCs (OR, 0.40; 95% CI, 0.18-0.86; *P* = .01). In addition, when PCPs were asked about whether they recommended 1 screening vs repeated or periodic screening, more than 40% recommended repeated or periodic screening for anemia in 65-year-old patients (132 of 324 [40.7%] for men and 133 of 324 [41.0%] for women) (Figure 1).

Because several guidelines recommend screening for anemia during pregnancy, an additional scenario of a 35-year-old pregnant woman was included to compare with the scenario of the 35-year-old woman who was not pregnant. For the pregnancy scenario, 105 of 325 PCPs (32.3%) reported they did not provide care to pregnant patients within their practices, deferring care of pregnant patients to obstetrics and gynecology specialists. Of the 220 PCPs who reported they would see pregnant patients in their practice, 188 (85.5%) reported they would screen a pregnant patient for anemia during her first trimester, as recommended in guidelines from the Centers for Disease Control and Prevention and the American College of Obstetricians and Gynecologists but not in the 2015 US Preventive Services Task Force updated guidelines.^15,16,17^

### Interpretation of Laboratory Studies

To assess PCPs’ choices for additional laboratory tests to be performed for evaluation of IDA, respondents were presented with a hypothetical scenario of a 65-year-old man reporting generalized fatigue who had a hemoglobin level of 10.4 g/dL (to convert to g/L, multiply by 10.0) and mean corpuscular volume of 72.3 fL (to convert to μm^3^, divide by 1.0). Measurement of ferritin level, the most specific marker of iron deficiency, was the most common laboratory test recommended by respondents (302 of 325 [92.9%]), followed by total iron-binding capacity and transferrin saturation (278 of 325 [85.5%]).

**Figure 2.**
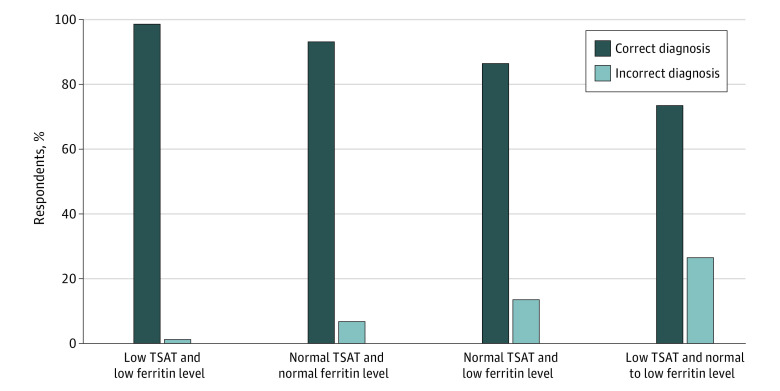
Interpretation of Iron Studies for Diagnosis of Iron Deficiency Anemia in 4 Scenarios With Different Combinations of Ferritin Levels and Transferrin Saturation (TSAT) Details of the scenarios are given in the Questionnaire Design subsection of the Methods section.

To assess PCPs’ interpretation of iron laboratory studies, respondents were presented with a series of laboratory values and asked to determine whether the anemia resulted from iron deficiency or an alternate cause. For scenarios in which ferritin level and transferrin saturation were concordantly low or normal, respondents correctly identified the cause with more than 93% accuracy: 321 of 325 respondents (98.8%) correctly attributed anemia to iron deficiency in the presence of a low transferrin saturation (6%) and a low ferritin level (11 ng/mL), and 303 of 325 (93.2%) correctly attributed anemia to an alternate cause in the presence of a normal transferrin saturation (30%) and a normal ferritin level (150 ng/mL) (Figure 2). However, in scenarios of discordant ferritin and transferrin saturation values, diagnostic accuracy decreased. For example, in a scenario with a normal transferrin saturation (25%) but low ferritin level (6 ng/mL), 44 of 325 respondents (13.5%) misidentified the cause as something other than iron deficiency even though the likelihood ratio for iron deficiency with a ferritin level less than 15 ng/mL is 51.9.^18^ In a scenario with a low transferrin saturation (2%) but borderline low ferritin level (40 ng/mL), 86 of 325 respondents (26.5%) did not consider IDA as the cause even though the likelihood ratio for iron deficiency with a transferrin saturation less than 5% is 10.5.^18^ In that scenario, 239 of 325 respondents (73.5%) correctly identified the scenario as IDA. There were no statistically significant differences in correct interpretation of these scenarios by academic affiliation status, gender, stage in clinical practice, or practice of ordering ferritin level measurement for anemia evaluation.

### Diagnostic Evaluation of IDA

**Figure 3.**
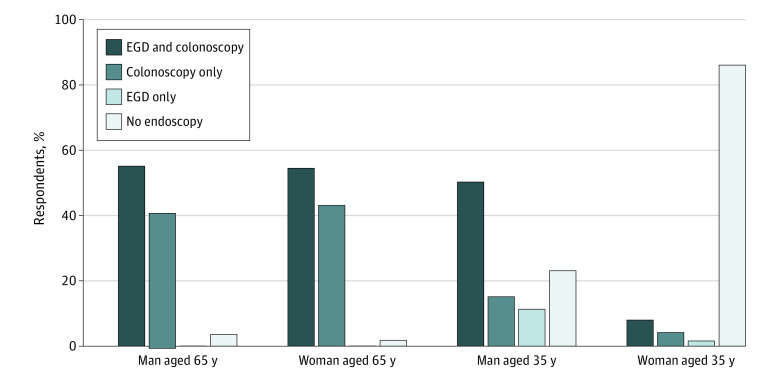
Initial Diagnostic Evaluation Recommended by Respondents for Patients With New-Onset Iron Deficiency Anemia and Negative Celiac Serologic Test Results by Age and Sex EGD indicates esophagogastroduodenoscopy.

Of 325 respondents, 316 (97.2%) reported that PCPs, not specialists, were primarily responsible for management of IDA within their practice settings. Respondents were asked about which tests they would obtain in a series of scenarios with new-onset IDA and negative celiac serologic test results in men and women aged 35 or 65 years. For the 65-year-old patients, nearly all respondents recommended initial GI endoscopic evaluation with colonoscopy and/or EGD (318 [97.9%] for women and 314 [96.6%] for men). Most respondents recommended performing both EGD and colonoscopy for 65-year-old patients (170 of 312 [54.5%] for women and 168 of 305 [55.1%] for men) (Figure 3). However, some recommended colonoscopy alone (without EGD) for initial evaluation (135 of 312 [43.3%] for women and 124 of 305 [40.7%] for men), which could lead to a potential diagnostic delay and additional sedation episode if upper GI pathologic findings were present or colonoscopy findings were normal. There were no statistically significant differences in the decision to perform EGD and colonoscopy for 65-year-old patients based on respondents’ academic affiliation status, gender, stage in clinical practice, practice type, or availability of open or direct access EGD and colonoscopy (without prior GI consultation).

Although respondents reported managing IDA in 65-year-old men and women similarly, they used a less invasive approach with 35-year-old patients. Of 325 PCPs, 314 (96.6%) recommended initial endoscopy for a 65-year-old man, but only 250 of 323 (77.4%) recommended initial endoscopic evaluation for a 35-year-old man (Figure 3). In addition, respondents reported differences in the use of GI endoscopy for 35-year-old patients based on the patient’s sex. For example, a 35-year-old man was more likely to undergo initial endoscopic evaluation with an EGD and colonoscopy than was a 35-year-old woman (OR, 5.68; 95% CI, 1.89-17.03; *P* = .001). However, even for a 35-year-old-man, there was a potential for a diagnostic delay, with 73 of 323 respondents (22.6%) recommending against initial endoscopic assessment. There were no statistically significant differences in recommendations for diagnostic endoscopy for a 35-year-old man based on respondents’ academic affiliation status, gender, stage in clinical practice, or practice type. The respondents were least likely to recommend any initial endoscopic evaluation for a 35-year-old woman (45 of 314 [14.3%]). For a 35-year-old woman, using empirical iron supplementation first was recommended by 264 of 312 respondents (84.6%). Respondents’ academic affiliation status, gender, stage in clinical practice, and practice type were not significantly associated with a decision to pursue diagnostic endoscopy for a 35-year-old woman.

## Discussion

In this nationally representative survey study of internal medicine PCPs, we found substantial variation in the management of anemia. Specifically, we found self-reported evidence of both overuse of screening CBCs (76.9% of respondents) and underuse of bidirectional endoscopy in patients with IDA. Certain knowledge gaps also existed in the interpretation of discordant laboratory test results, which may contribute to diagnostic errors. Specifically, 26.5% of respondents misdiagnosed a scenario of IDA as anemia with an alternate cause despite a low transferrin saturation (2%) and a borderline low ferritin level (40 ng/mL). Several subtle but important nuances in interpretation of these studies may contribute to errors, including the high specificity of a low ferritin level (98% specific for iron deficiency with a ferritin level ≤12 ng/mL),^24^ the potential for the ferritin level to be normal or elevated in inflammatory states when a patient is iron deficient,^2,18^ and the importance of measuring serum iron level in a fasting state to avoid potential confounders of dietary or supplemental iron.^25,26^ Given the relatively high baseline prevalence of anemia of 5.6% in the US,^13^ incorrect interpretation of these laboratory results may lead to a substantial number of missed or delayed diagnoses of IDA and, by extension, potential delays in diagnosis of GI tract malignant neoplasm.

Our findings are in line with those of older studies on this topic. Prior work has found limited clinical benefit associated with the common practice of obtaining routine CBCs for hospitalized patients^27,28^ and outpatients.^29,30,31^ But in a repeated survey study of family medicine PCPs, the frequency of routine CBCs remained persistently high between 1978 and 2004, with 67% of respondents reporting that they would obtain a CBC for a 55-year-old woman in the most recent survey.^32^ Although overuse is a complex phenomenon, these data suggest that routine laboratory assessments are ingrained within medical practice. In this context, it is notable that, to our knowledge, there are no guidelines (eg, from Choosing Wisely or other organizations) explicitly recommending against screening for anemia in outpatients.^17,33,34^ In contrast, Choosing Wisely explicitly recommends against routine or repeated CBCs for hospitalized patients.^33,35^ Although the 2015 US Preventive Services Task Force guidelines determined that there was insufficient evidence to judge the benefits or harms of screening for anemia during pregnancy,^17,34^ there is a potential for adverse neurodevelopmental outcomes during pregnancy when the woman has iron deficiency^36^; multiple international guidelines have recommended screening for anemia during pregnancy despite limited evidence.^37^ In this context of absent or conflicting recommendations, most respondents in the present survey study nonetheless reported they would obtain screening CBCs for patients who were pregnant or not pregnant. Prior work^38,39^ has also reported underuse of bidirectional endoscopy, although not to the extent found in our study. Although previous guidelines recommended bidirectional endoscopy for men and postmenopausal women,^11^ subsequent American Gastroenterological Association guidelines, published after this survey was conducted, recommend bidirectional endoscopy regardless of menopausal status for those without an alternate explanation for IDA.^12^ Despite these recommendations, some physicians’ selection of colonoscopy instead of combined EGD and colonoscopy for initial evaluation of IDA may be reflective of lack of access. For example, in some practice settings, insurance reimbursements may disincentivize combined EGD and colonoscopy, and physicians may not offer the combined procedures in a single appointment.^38,39^ However, a serial diagnostic testing approach may lead to increased costs and require that patients undergo a second episode of sedation. In addition, if there is a substantial time interval between the colonoscopy and the EGD, there is the theoretical potential for a delay in diagnosis (although serial testing was not directly assessed in this survey study).

Given the potential consequences of missed or underevaluated IDA and the complexities of test interpretation, clinicians may benefit from tools within the electronic health record to help improve diagnosis and reduce medical errors. For example, instead of displaying individual laboratory values in isolation, a computer-assisted diagnostic tool could be developed to display an individualized interpretation of the most likely cause of anemia in a patient. This would allow for more precise interpretation of laboratory studies for individuals, such as interpreting iron studies for a patient with inflammation. In addition, automated “trigger tools,” or detection algorithms for abnormal laboratory patterns, may help identify cases of new-onset anemia that had been potentially missed by busy clinicians.^5^ After abnormalities are identified by these tools, a common set of clinical actions could be presented (eg, for ordering endoscopic testing) to help facilitate obtainment of appropriate tests and minimize additional tasks for clinicians. Reducing unnecessary CBC ordering through education or displaying cost of the laboratory testing alone is unlikely to be sufficient,^40^ but a multilevel intervention incorporating education, price information, feedback, and financial incentives has demonstrated success in an inpatient setting.^41^

### Limitations

This study has limitations. Respondents may have answered questions based on what they believed was the correct evidence-based practice rather than what was reflective of their actual clinical practice, potentially introducing response bias. Other limitations include the possibility that participants in the ACP Insiders Panel were more engaged with the organization and thus more aware of current evidence-based practices. Both of these biases would suggest that the knowledge gaps identified in our study are underestimates of those seen in actual practice. The results may not apply to other professionals who provide primary care, such as family medicine physicians or advanced practice physicians. A potential for nonresponse bias exists, and those who did not respond may have been less knowledgeable about the topic or may have differed in other ways related to the outcomes of interest. However, we attempted to mitigate this bias through systematic efforts to increase responses. In addition, we analyzed available demographic data for respondents and nonrespondents and found no significant differences in age, geographic location, or career stage; however, respondents were more likely to be White and male than were nonrespondents. Survey methods may not be as well suited for the study of actual practice behaviors as other methods. However, our survey allowed us to indirectly assess physician knowledge and behavior with uniform, ideal clinical scenarios, which is not possible when secondary data are used.

## Conclusions

In this survey study, PCPs’ self-reported testing practices for anemia suggest overuse of screening laboratory tests, misinterpretation of iron studies, and underuse of bidirectional endoscopy in evaluation of new-onset IDA. These findings suggest that IDA, a common clinical condition and early sign of GI malignant neoplasm, is not uniformly understood, approached, and managed by practicing physicians in the US. Given the potential association with GI cancers and the increasing incidence of colorectal cancer in younger adults, thorough evaluation of IDA is important for preventing delays in diagnosis.^3,4,9^ Further work should inform the design and implementation of tools to improve diagnostic evaluation of IDA and reduce the risk of potential diagnostic errors.

## Notes

Supplement. **eAppendix.** Anemia Survey
