# Supplementary material for: Testing Practices, Interpretation, and Diagnostic Evaluation of Iron Deficiency Anemia by US Primary Care Physicians
Source: JAMA Netw Open. 2021 Oct 1;4(10):e2127827. doi: 10.1001/jamanetworkopen.2021.27827 (PMC8486982; doi:10.1001/jamanetworkopen.2021.27827)
Supplement: Supplement. — eAppendix. Anemia Survey [file jamanetwopen-e2127827-s001.pdf]

Supplementary Online Content

Read AJ, Waljee AK, Sussman JB, et al. Testing practices, interpretation, and diagnostic evaluation of iron deficiency anemia by US primary care physicians. *JAMA Netw Open*. 2021;4(10):e2127827. doi:10.1001/jamanetworkopen.2021.27827

eAppendix. Anemia Survey

This supplementary material has been provided by the authors to give readers additional information about their work.

© 2021 Read AJ et al. *JAMA Network Open*.

eAppendix. Anemia Survey

1. **Which of the following best describes you at this time? (Choose one)**

Resident/fellow in training	Terminate
Completed training and active in medicine	
Fully retired/currently not working in medicine	Terminate

2. **What is your <u>primary</u> specialty area (i.e., the specialty area where you spend most of your time)? (Choose one)**

General internal medicine	
Hospitalist	Terminate
Geriatric subspecialist	Terminate
Other subspecialist (e.g., cardiologist, pulmonologist, etc.)	Terminate

3. **What percentage of your time is spent delivering primary care to your patients? (Choose one)**

None	Terminate
Less than 25%	
25% to 49%	
50% or more	

4. **Imagine that you are seeing a new patient in your clinic to establish care. Which of the following best describes your use of Complete Blood Count (CBC) testing for <u>detection of anemia</u> in the following type of patient?**

Type of Patient	Would <u>not</u> order a CBC	Would order a CBC once	Would order a CBC now and repeat periodically	Would not manage this patient in my practice
A 65 year old healthy MAN				
A 65 year old healthy WOMAN				
A 35 year old healthy MAN				
A 35 year old healthy WOMAN who is not pregnant				
A 35 year old healthy WOMAN who is pregnant and in her first trimester				

© 2021 Read AJ et al. *JAMA Network Open*.

5. **For the following patients, which best describes current screening recommendations for anemia?**

	Routine testing is <u>not</u> recommended	Routine testing is recommended
All adults greater than 18 years of age		
All pregnant patients		
All menstruating patients		
All adults greater than 50 years of age		

6. **Imagine that a 65 year old man comes to see you complaining of generalized fatigue with no other associated symptoms. His physical examination is unremarkable and his labs show a hemoglobin of 10.4 g/dL with a mean corpuscular volume (MCV) of 72.3 fl. No other studies are available. Based on the tests you typically order to evaluate suspected iron deficiency anemia, which of the following laboratory tests would you obtain at this visit for further work-up?**

	I would <u>not</u> order this lab	I would order this lab
Serum iron		
Total iron binding capacity (TIBC)/Transferrin		
Serum Ferritin		
Celiac serologies		

7. **If the results of lab testing demonstrate the following, does this represent iron deficiency anemia (IDA) or anemia of another cause?**

Hemoglobin (g/dL)	Iron/TIBC (transferrin Saturation)	Ferritin (ng/mL)	Definitely IDA	Probably IDA	Probably <u>not</u> IDA	Definitely <u>not</u> IDA
10.4	6%	11				
10.3	30%	150				
10.4	25%	6				
10.5	2%	40				

© 2021 Read AJ et al. *JAMA Network Open*.

8. Seeing a 65 year old healthy MAN in clinic who was found to have newly diagnosed IDA and negative celiac serologies. Based on THIS VISIT, which of the following would be your next step(s) in management?

	**I would <u>not</u> do this**	**I would do this**
Oral iron supplementation		
Obtain colonoscopy		
Obtain upper endoscopy (EGD)		

9. Seeing a 65 year old healthy WOMAN in clinic who was found to have newly diagnosed IDA and negative celiac serologies. Based on THIS VISIT, which of the following would be your next step(s) in management?

	**I would <u>not</u> do this**	**I would do this**
Oral iron supplementation		
Obtain colonoscopy		
Obtain upper endoscopy (EGD)		

10. Assume you are seeing a 35 year old healthy WOMAN (who is <u>not</u> pregnant) who presents with newly diagnosed IDA and negative celiac serologies. Based on THIS VISIT, which of the following would be your next step(s) in management?

	**I would <u>not</u> do this**	**I would do this**
Oral iron supplementation		
Obtain colonoscopy		
Obtain upper endoscopy (EGD)		

© 2021 Read AJ et al. *JAMA Network Open*.

11. **Assume you are seeing a 35 year old healthy MAN with newly diagnosed IDA and negative celiac serologies. Based on THIS VISIT, which of the following would be your next step(s) in management?**

	**I would not do this**	**I would do this**
Oral iron supplementation		
Obtain colonoscopy		
Obtain upper endoscopy (EGD)		

12. **At what age do you typically start screening for colorectal cancer in patients who are at average risk?**

Less than 40 years of age
40 – 44 years of age
45 – 49 years of age
50 – 60 years of age
Over 60 years of age

13. **Were you aware of the American Cancer Society's 2018 updated recommendations of when to start colorectal cancer screening?**

Yes -- it has changed my clinical practice
Yes -- it has not yet changed my clinical practice
No

14. **Do you agree or disagree that generally you are reluctant to suggest watching and waiting for your patients because you are worried about missing something important?**

Strongly agree
Agree
Disagree
Strongly disagree
Not sure

© 2021 Read AJ et al. *JAMA Network Open*.

15. Do you agree or disagree that generally in our healthcare system too many labs and diagnostic procedures are performed?

Strongly agree
Agree
Disagree
Strongly disagree
Not sure

16. Which of the following best describes your main practice setting? (Choose one)

Single specialty office-based practice
Multispecialty office-based practice
Hospital-based practice
Free standing ambulatory care or urgent care center
Medical school/academic medical center/University
US government clinic (including VA/military)
Institution (prison, nursing home, long term care facility, student health)
Other (Specify: ____)

17. Which of the following "open" or "direct" access procedures are you able to obtain in your practice setting; i.e., order a procedure without an intervening gastroenterology clinic visit? (Choose all that apply)

Video Capsule Endoscopy
Upper Endoscopy (EGD)
Colonoscopy
None – no open/direct access procedures are available (Exclusive response)

18. In your practice setting, who is primarily responsible for managing iron deficiency anemia? (Choose one)

Myself (the primary care physician)
Hematologist
Gastroenterologist
OB-GYN
Nephrologist

© 2021 Read AJ et al. *JAMA Network Open*.

19. Which of the following best describes the community in which your practice is located?

Urban
Suburban
Rural

20. How would you describe where you provide the majority of your patient care? (Choose one)

All outpatient
Primarily outpatient with some inpatient
Primarily inpatient with some outpatient
Equal outpatient and inpatient

21. For how many years (since completing your residency or fellowship) have you been in clinical practice?

_________ Years

22. Are you currently board certified in Internal Medicine?

Yes
No

23. Are you currently affiliated with a medical school as a clinical instructor or other faculty appointment?

Yes
No

24. What is your gender?
Male
Female
Transgender
Prefer not to answer

25. Lastly, what is your race?
Asian
American Indian or Alaskan Native
Black or African American
Native Hawaiian or Pacific Islander
White
Other (Specify: ____)

© 2021 Read AJ et al. *JAMA Network Open*.
